# Preparing families and institutions to manage anaphylaxis

**DOI:** 10.1186/2045-7022-1-S1-S60

**Published:** 2011-08-12

**Authors:** Susanne Halken

**Affiliations:** 1 Hans Christian Andersen Children's Hospital, Odense University Hospital, Odense, Denmark

## 

Anaphylaxis is a pediatric emergency, but most cases occur in the community outside a healthcare setting. It is therefore essential that the families, schools, nurseries and other child care professionals understand how to avoid, recognise and manage severe allergic reactions. Food allergy is the most common cause of anaphylactic reactions in children. If children are to avoid the food allergens, both their parents and the children themselves need to know exactly what food products to avoid and how to do it. This knowledge is best taught by a dietician with expertise in pediatric allergy. It is also important that the child’s other carers are trained. In a recent study it has been shown that parents to children with severe food allergy had a very poor understanding of how to avoid further contact with the trigger food. After education by an experienced dietician these families’ knowledge increased significantly from 55% to 70%. Unfortunately, even with the best education, children are going to come into contact with foods they are allergic to. The child and their family therefore need to know how to recognise an allergic reaction and appropriately deal with it. This is facilitated with the use of personalised management plan that take into account the child’s personal risk of anaphylaxis and coexistent medical problems. This plan should include both prescription and training of the child and family in self-administration of adrenalin. It has been shown that at initial presentation to a tertiary clinic, only half of the families understood how to manage an anaphylactic reaction and they did not know when to carry an EpiPen, how to use it or when to call an ambulance. After education by a skilled pediatric nurse their overall understanding of how to manage allergic reactions and how to use an EpiPen increased to around 95%. Thus, there is a need for education and training of families with children at risk for anaphylaxis, and studies indicate that such educational programmes may increase both knowledge and abilities of the families to avoid risk situations and to manage emergencies.

